# Can omalizumab be an alternative treatment for non-atopic severe asthma? A real-life experience with omalizumab

**DOI:** 10.5578/tt.20239904

**Published:** 2023-03-10

**Authors:** F.E. Günaydın, D. Ediger, M. Erbay, G. Pekbak

**Affiliations:** 1 Department of Allergy and Immunology, Bursa Uludağ University Faculty of Medicine, Bursa, Türkiye

**Keywords:** asthma, immunoglobulin E, biologic agents, omalizumab

## Abstract

**ABSTRACT:**

Can omalizumab be an alternative treatment for non-atopic severe asthma? A real-life experience with omalizumab

**Introduction:**

Omalizumab, a humanized monoclonal anti-IgE antibody, has largely demonstrated its efficacy in severe allergic asthma. There are limited data about the effectiveness of omalizumab in patients with non-atopic severe persistent asthma. In this study, we aimed to determine the effect of omalizumab in patients with non-atopic severe asthma and compare the data obtained with those in patients with allergic severe asthma.

**Materials and Methods:**

This study was an observational, retrospective, tertiary single-center study that assessed and compared the clinical outcome of adult patients with severe asthma (165 atopic and 41 non-atopic) who have been on omalizumab for one year or longer between January 2008 and January 2020. Effectiveness was assessed by considering symptom scores (GINA symptom control score), daily systemic corticosteroids (SCS) dosage, blood eosinophil counts, pulmonary function, and number of severe exacerbations and hospitalizations within the last one year.

**Results:**

Omalizumab exhibited significant improvement in the clinical status of non-atopic asthma patients as measured by GINA symptom score [decreased from 3.77 ± 0.63 to 1.36 ± 1.27 (p< 0.001)], the number of emergency room visits for asthma [decreased from 11.25 ± 14.69 to 0.25 ± 0.55 (p< 0.001)], and the number of hospitalizations [decreased from 1.17 ± 2.87 to 0.14 ± 0.36 (p= 0.036)]. These results were not significantly different from those obtained in allergic asthma patients. FEV_1_ improved significantly from 2.08 ± 0.86 to 2.14 ± 0.84 (p= 0.041) and oral corticosteroid doses decreased significantly from 1.67 ± 7.49 to 0.46 ± 2.74 (p= 0.015) in the only atopic group.

**Conclusion:**

Omalizumab, which is a proven and effective treatment option for allergic asthma, may also be an efficacious alternative option in non-atopic severe asthma.

## Abstract (tr)

**ÖZ:**

Omalizumab atopik olmayan ağır astımda alternatif bir tedavi olabilir mi? Omalizumab ile gerçek yaşam deneyimi

**Giriş:**

Humanize monoklonal bir anti-IgE antikoru olan omalizumab, ağır alerjik astımda etkinliğini büyük ölçüde göstermiştir. Non atopik ağır astımı olan hastalarda omalizumabın etkinliğiyle ilgili sınırlı veri vardır. Bu çalışmada non atopik ağır astımda omalizumabın etkisini belirlemeyi ve alerjik astımlı olgularla karşılaştırmayı amaçladık.

**Materyal ve Metod:**

Bu retrospektif gözlemsel çalışmada üçüncü basamak tedavi merkezinde Ocak 2008 ile Ocak 2020 arasında bir yıl veya daha uzun süredir omalizumab kullanan ağır astımlı (165 alerjik ve 41 atopik olmayan) erişkin hastaların klinik sonuçları değerlendirildi. Etkinlik; GINA semptom skoru, günlük sistemik kortikosteroid (SK) dozu, kan eozinofil sayıları, akciğer fonksiyonu ve son bir yıldaki ciddi alevlenmeler ve hastaneye yatış sayısıyla değerlendirildi.

**Bulgular:**

Omalizumab ile non-atopik astım hastalarında GINA semptom skoru 3,77 ± 0,63’ten 1,36 ± 1,27’ye (p< 0,001), astım için acil servis ziyaret sayısı 11,25 ± 14,69’dan 0,25 ± 0,55’e (p< 0,001), yıllık hastaneye yatış sayısı 1,17 ± 2,87’den 0,14 ± 0,36’ya (p= 0,036) gerilemiştir. Bu sonuçlar alerjik astımlı hastalar ile benzerdi, iki grup arası fark istatistiksel olarak anlamlı değildi. Alerjik astımlı grupta farklı olarak FEV_1_ 2,08 ± 0,86 lt’den 2,14 ± 0,84 lt’ye yükseldi (p= 0,041) ve oral kortikosteroid dozları anlamlı olarak 1,67 ± 7,49 mg’dan 0,46 ± 2,74 mg’a geriledi (p= 0,015).

**Sonuç:**

Alerjik astımda kanıtlanmış ve etkin bir tedavi seçeneği olan omalizumab, non-atopik ağır astımda da etkili bir alternatif olabilir.

## Introduction

Severe asthma is a subset of difficult-to-treat asthma that remains uncontrolled despite adherence to maximal optimized therapy and treatment of contributory factors, or that worsens when high-dose treatment is decreased. It affects about 3.7% of all asthma patients and is associated with high morbidity, mortality, and economic burden ( 4 ). Severe asthma has been divided into two pathogenic variants, atopic and non-atopic asthma, based on the presence or absence of clinical allergic reaction and in vitro/in vivo IgE response to specific aeroallergens. Despite many similarities between atopic and non-atopic asthma with regard to cellular and molecular immunopathology in the bronchial mucosa, the nonatopic form of the disease is characterized by a higher degree of severity ( 4 , 5 , 6 ).

Omalizumab (Xolair; Novartis, Switzerland), which is a humanized anti-IgE monoclonal antibody (mAb) that selectively binds to human IgE and prevents the binding of IgE to its receptors, is an add-on treatment option effective in the treatment of patients with severe allergic asthma ( 4 ). Up to 50% of patients with severe asthma are non-atopic and previous studies suggested that off-label uses of omalizumab in nonatopic asthma could be an effective way to control symptoms and increase the quality of life ( 7 , 8 , 9 ).

Omalizumab was approved in Türkiye in 2008 and our tertiary referral asthma center has been using this drug in atopic and non-atopic severe asthma patients.

Studies on the off-label use of omalizumab in nonatopic asthma are limited in the literature (7-9^4^). Hence, we aimed to assess the clinical efficacy of omalizumab in real-world setting, in a population of 41 severe non-atopic asthma patients, and compare it with 165 severe allergic asthmatics, who received the same treatment.

## MATERIALS and METHODS

### Study Design

This retrospective clinical study was performed in the Department of Allergy and Immunology of the Bursa Uludağ University Medical Faculty Hospital. The study was approved by Uludağ University Faculty of Medicine Clinical Research Ethics Committee (Decision no: 2020/10-04, Date: 10.06.2020).

### Subjects

The medical records of adult patients with severe asthma who were treated with omalizumab for >12 months between January 2008 and January 2020 and benefits were retrospectively analyzed. All patients were divided into two main groups according to their perennial allergy status as allergic and non-atopic asthmatics. Non-atopics were identified based on negative skin testing to house dust mites (Dermatophagoides pteronyssinus, Dermatophagoides farinae), mold (Fusarium, Alternaria), cat, dog, and cockroach. The non-atopic patient group also had negative serum-specific IgE to house dust mites (Dermatophagoides pteronyssinus, Dermatophagoides farinae).

Inhaler technique, adherence, and comorbidities were investigated in all the patients. The omalizumab (Xolair; Novartis, Switzerland) dose was calculated according to body weight and total immunoglobulin (Ig)E level. Non-atopic patients took omalizumab off-label. All patients are still receiving omalizumab and are regularly followed up in our clinic.

### Measurements

Demographic characteristics and clinical features of asthma (time since diagnosis, total IgE level, allergy status documented by skin prick test (SPT) for common aeroallergens) were recorded from patients’ documents.

The diagnosis of asthma was made using the clinical history and by demonstrating objective measures of reversible airway obstruction [forced expiratory volume in one second (FEV_1_)< 80% and FEV_1_/forced vital capacity (FVC)< 70% with an improvement in FEV_1_ 12% and 200 mL after 400 mcg of salbutamol or average daily diurnal PEF variability> 10% over two weeks)]. In the absence of reversible airflow limitation in PFT, asthma was diagnosed based on average daily diurnal PEF variability.

The SPT was performed using a standard commercial extract panel (Alk-Abello, Lincoln Diagnostics, Dallas, TX, USA), consisting of 17 aeroallergens (Dermatophagoides pteronyssinus, D. farinae, Acarus siro, grass mix, grass-rye mix, trees mix, oak, birch, weed mix, wheat, olive, latex, dog, cat, cockroach, Alternaria, Fusarium). Skin prick test was conducted according to the international guidelines as a single test on two forearms with lancets and standardized allergens by the same trained nurse. Histamine hydrochloride (10 mg/mL) and 0.9% saline were applied as positive and negative controls, respectively. The wheel diameter was measured after 20 minutes and reported in mm. A skin reaction of ≥3 mm produced by the negative control on the SPT was considered a positive reaction. Serum-specific IgE for house dust mites (Dermatophagoides pteronyssinus, Dermatophagoides farinae) evaluated for skin prick test negative patients, negative cases classified nonatopic group.

For every patient, we recorded baseline and current results for the following variables: blood eosinophil counts, symptom control scores forced expiratory volume in one second (FEV_1_) values, medications, and the number of exacerbations that required systemic corticosteroids (SCS) for at least three days (outpatient clinic visit, ER admission, and hospitalization) within the last year. SCS and inhaled corticosteroids (ICS) doses were calculated as their methyl-prednisolone and budesonide equivalents, respectively. GINA symptom control tool, which assesses daytime symptoms, reliever treatment use, night waking, and activity limitation in the past four weeks, was used to evaluate symptom control. GINA symptom control scores were grouped into wellcontrolled (score 0), partly-controlled (score 1-2), or uncontrolled (score 3-4) ( 4 ).

### Outcomes

The effectiveness of omalizumab was assessed over the treatment period through decreases in symptom score and improvements in pulmonary function tests obtained from patients’ exacerbation-free periods, as well as a reduction in exacerbations, emergency visits, and hospitalizations, all assessed by at least three doctors who were all authors of this manuscript. An exacerbation has been defined as the acute deterioration of symptoms and lung functions from the usual status of the patient that requires unplanned medical management and an increase in daily medications.

### Analysis

The data were analyzed using the Statistical Package for Social Sciences (SPSS) version 23 software (IBM Corp., Armonk, NY, USA). The distribution of the data was analyzed by using the Kolmogorov-Smirnov test. Numeric values with normal dispersion were expressed as mean ± standard deviation (SD). Paired sample t-test was used to test the differences between pre- and post-treatment. Between-group comparisons were performed using Mann-Whitney U test, independent sample t-test, or Chi-square test, as relevant. A p-value of less than 0.05 was considered statistically significant.

## RESULTS

### Demographic and Baseline Characteristics

A total of 206 patients with severe asthma (165 allergic and 41 non-atopic) were included in this study with a mean age of 51.71 ± 13.78 years. The mean duration of asthma was 14.62 ± 11.32 years for the allergic group, and 19.08 ± 17.27 years for the non-atopic group (p> 0.05). Obesity was common in both groups. The demographic and baseline characteristics are compared in Table 1 .

**Table 1:** Demographic and baseline clinical characteristics before omalizumab (continue)

	Allergic (n= 165)	Non-atopic (n= 41)	Total (n= 206)	p
Age, years ± SD				
18-29 years	17 (10.3)	2 (4.9)	17 (8.5)	0.214
30-49 years	56 (33.9)	11 (26.8)	65 (32.5)	
50-65 years	68 (41.2)	17 (41.5)	77 (38.5)	
>65 years	24 (14.5)	11 (26.8)	41 (20.5)	
Female gender, n (%)	144 (87.3)	37 (90.2)	177 (88.5)	0.791
BMI				
Under/Normal weight	39 (23.9)	6 (14.6)	45 (22.7)	0.427
Overweight	40 (24.5)	12 (29.3)	48 (24.2)	
Obese subjects	84 (51.5)	23 (56.1)	105 (53.0)	
Smoking status, n (%)				
Smokers	105 (66.0)	0 (0.0)	13 (6.7)	0.142
Ex-smokers	14 (8.8)	10 (25.0)	49 (25.4)	
Nonsmokers	40 (25.2)	30 (75.0)	131 (67.9)	
Pack year	16.65 ± 17.01	9.45 ± 12.08	14.79 ± 15.57	0.207
Comorbidities, n (%)				
Chronic rhinitis	149 (90.3)	32 (78.0)	177 (88.5)	0.057
AERD	18 (11.3)	2 (4.9)	20 (10.3)	0.379
Urticaria	16 (10.1)	3 (7.3)	19 (9.8)	0.769
GER	40 (25.3)	14 (34.1)	52 (26.9)	0.257
Nasal Polyps	18 (11.3)	1 (2.4)	19 (9.7)	0.131
Sinusitis	75 (45.5)	22 (53.7)	93 (46.5)	0.346
Bronchiectasis	16 (9.7)	6 (14.6)	21 (10.5)	0.397
ABPA	3 (1.8)	0 (0.0)	3 (1.5)	>0.999
CSS	2 (1.2)	0 (0.0)	2 (1.0)	>0.999
Onset age of asthma, years	35.75 ± 14.18	14.80 ± 18.34	35.71 ± 15.09	0.722
Duration of asthma, years	14.62 ± 11.32	19.08 ± 17.27	15.56 ± 12.93	0.128
Allergen sensitization status, n (%)				
Mono-sensitized	69 (43.4)	2 (4.9)	67 (34.5)	<0.001
Poly-sensitized	90 (56.6)	0 (0.0)	87 (44.8)	<0.001
Total IgE, IU/mL	269.07 ± 410.19	191.83 ± 474.406	257.10 ± 428.83	0.297
Omalizumab monthly dose, mg	413.64 ± 301.89	318.29 ± 284.99	395.25 ± 297.42	0.069
Duration of omalizumab treatment, n (%)				
12-24 months	34 (20.7)	2 (4.9)	36 (18.1)	0.001
24-36 months	24 (14.6)	6 (14.6)	30 (15.1)	
36-48 months	50 (30.5)	12 (29.3)	62 (31.2)	
48-60 months	11 (6.7)	12 (29.3)	22 (11.1)	
>60 months	45 (27.4)	9 (22.0)	49 (24.6)	
Serum eosinophil count, cells/mL	400.01 ± 455.78	405.37 ± 396.722	401.11 ± 443.36	0.945
GINA symptom score	3.52 ± 0.9	3.77 ± 0.63	3.58 ± 0.85	0.047
Controlled	21 (14.5)	2 (5.1)	21 (11.7)	0.172
Uncontrolled	124 (85.5)	37 (94.9)	158 (88.3)	
Emergency room visits for asthma	7.96 ± 14.71	10.46 ± 14.37	8.29 ± 14.47	0.340
Number of hospitalizations within the last 12 months	0.75 ± 1.86	1.11 ± 2.81	0.79 ± 2.06	0.350
SCS use within the last 12 months	5.79 ± 9.6	9.31 ± 13.94	6.29 ± 10.28	0.143
FEV_1_/FVC	75.86 ± 14.41	75.44 ± 12.66	75.29 ± 12.21	0.822
FEV_1_ lt	2.09 ± 0.85	1.85 ± 0.88	2.05 ± 0.86	0.155
FEV_1_ (% of predicted)	83.1 ± 25.86	78.71 ± 30.68	82.38 ± 26.86	0.415
FVC lt	2.73 ± 0.93	2.50 ± 1.00	2.69 ± 0.95	0.254
FVC (% of predicted)	92.13 ± 22.53	83.72 ± 28.61	90.53 ± 23.93	0.089
ICS dose	1556.69 ± 178.77	1531.71 ± 198.04	1554.64 ± 176.33	0.437
SCS dose	1.63 ± 7.40*	0.51 ± 2.56^ý^	1.43 ± 6.77	0.343
LABA	158 (100)	40 (97.6)	190 (97.9)	-
Montelukast	154 (98.1)	40 (97.6)	190 (97.9)	1.000
Theophylline	49 (31.2)	21 (51.2)	69 (35.6)	0.017
LAMA	28 (17.9)	6 (14.6)	34 (17.6)	0.617

*: 11 patients had received SCS, ý: Three patients had received SCS.

There were no significant differences between the comorbidities of the allergic group and the nonatopic group. All patients were on high-dose ICS (min: 1000 μg BDP/day, max: 2000 μg, BDP/day) plus LABA. The frequency of pre-omalizumab theophylline use in non-atopic asthmatics was higher than in the allergic group (p< 0.05) ( Table 1 ).

### Assessment of Clinical Response, Pulmonary Function Test, and Serum Eosinophil Count After Omalizumab

After omalizumab, the number of ER visits for asthma, steroid use, and hospitalization in the previous 12 months decreased statistically significantly in both allergic and non-atopic asthma groups (p< 0.05) ( Table 2 ).

GINA symptom score was significantly decreased in both groups after omalizumab (p< 0.05). The mean ICS dose was not significantly changed in both groups but the SCS dose was significantly decreased in allergic asthmatics. Mean FEV_1_ lt, FEV_1_% and FVC lt improved significantly in the allergic group after omalizumab (p< 0.05). FVC% is improved significantly in both allergic and non-atopic groups (p< 0.05) ( Table 2 ).

**Table 2:** Comparison of the pre-omalizumab and post-omalizumab eosinophil counts, symptom scores, exacerbation rates, pulmonary function measurements, ICS and SCS doses, and frequency of montelukast, theophylline, and LAMA

	Allergic (n= 165)	Non-atopic (n= 41)
	Pre omalizumab	Post omalizumab	p	Pre omalizumab	Post omalizumab	p
Emergency room visits for asthma	7.28 ± 13.07	0.41 ± 1.09	<0.001	11.25 ± 14.69	0.25 ± 0.55	<0.001
Number of hospitalizations within the last 12 months	0.76 ± 1.88	0.08 ± 0.43	<0.001	1.17 ± 2.87	0.14 ± 0.36	0.036
Steroid use within the last 12 months	5.65 ± 9.56	0.58 ± 2.79	<0.001	10 ± 14.3	0.53 ± 0.97	<0.001
GINA symptom score	3.52 ± 0.90	1.13 ± 1.19	<0.001	3.77 ± 0.63	1.36 ± 1.27	<0.001
FEV_1_/FVC	74.91 ± 12.30	74.8 ± 11.54	0.879	76.62 ± 14.05	73.38 ± 11.15	0.084
FEV_1_ ,lt	2.08 ± 0.86	2.14 ± 0.84	0.041	1.85 ± 0.88	1.95 ± 0.92	0.314
FEV_1_, % of predicted	81.56 ± 25.93	85.79 ± 24.18	0.001	78.71 ± 30.68	84 ± 27.82	0.142
FVC, lt	2.69 ± 0.92	2.79 ± 0.88	0.004	2.50 ± 1.00	2.67 ± 1.14	0.080
FVC, % of predicted	90.38 ± 22.41	95.5 ± 19.19	<0.001	83.72 ± 28.61	96.61 ± 23.86	0.017
ICS dose, mg	1556.41 ± 179.31	1528.21 ± 250.88	0.205	1531.71 ± 198.04	1570.73 ± 138.28	0.253
SCS dose, mg	1.67 ± 7.49	0.46 ± 2.74	0.015	0.54 ± 2.62	0.1 ± 0.64	0.295
Serum eosinophil count, cells/mL	421.54 ± 477.56	340.56 ± 335.52	0.006	411.13 ± 398.64	363 ± 370.2	0.135

Serum eosinophil count decreased significantly in the allergic group (n= 117; before omalizumab, 421.54 ± 477.56 cells/μL; after omalizumab, 340.56 ± 335.52 cells/μL; p< 0.05). In the non-atopic group serum eosinophil count did not decrease significantly, from 411.13 ± 398.64 cells/μL to 363 ± 370.2 cells/ μL ( Table 2 ).

When the changes in eosinophil counts, symptom scores, exacerbation rates, pulmonary function measurements, and ICS and SCS doses after omalizumab in allergic and non-atopic severe asthmatics were compared, no significant difference was found between the groups (p> 0.05) ( Table 3 ).

## DISCUSSION

Our findings supported the clinical efficacy of omalizumab in non-atopic severe asthmatic patients in real-world settings. Omalizumab is indicated for patients with severe allergic asthma, however, there are currently few therapeutic options for severe non-atopic asthmatics, therefore many physicians prescribe it off-label. In our study, the number of emergency room visits for asthma, hospitalization number in the last 12 months, and steroid use in the last 12 months significantly decreased and GINA symptom scores increased in both allergic and non-atopic asthmatics. Pulmonary function values (mean FEV_1_ lt, FEV_1_% and mean FVC lt) improved significantly in the allergic group after omalizumab. When allergic and non-atopic groups were compared in terms of changes in these clinical parameters after omalizumab, there were no significant differences found between groups.

In the literature, there were limited studies about the use of omalizumab in severe non-atopic asthmatics. The effectiveness of omalizumab in patients with non-atopic severe asthma was shown as similar to that in allergic patients with severe asthma, characterized by improvements in pulmonary functions, asthma symptoms, reductions in exacerbations and hospitalizations, and days off sick from education/employment. It was also associated with significant improvements in lung function, asthma-specific and generic patient-reported outcomes (PROs), and the number of patients in employment ( 7, 8, 9, 10, 11 ). Considering all the studies, it should be pointed out that only one study compared omalizumab in severe allergic and non-atopic asthmatics that included 29 severe non-atopic asthmatics and 266 severe allergic asthmatics ( 11 ). Therefore, as a tertiary asthma center in the Southern Marmara region, we wanted to share our experience with omalizumab in both groups with available data

In our study, we found significantly important increases in GINA scores in both allergic and nonatopic groups after omalizumab, on the other hand, there were no significant differences between groups. Sözener et al. observed significant increases in ACT scores in 13 severe non-atopic asthmatic patients in the first two years after using omalizumab ( 8 ). Additionally, in a post-hoc sub-analysis of the FENOMA study, Campo et al. observed significant increases in ACT scores in 80 severe non-atopic patients in the first year follow-up after omalizumab ( 9 ). There is only one real-world multicenter study in the literature which included patients who received omalizumab for severe non-atopic and allergic asthma, where an ACT score of >19 was found in 17% of non-atopic patients at one year versus 29.3% in allergic asthma patients; and there were no significant differences between groups ( 11 ).

**Table 3:** Comparison of the differences between groups

Differences	Allergic	Non-atopic	p
Serum eosinophil count, cells/mL	80.98 ± 341.97	48.13 ± 194.33	0.571
GINA symptom score	2.39 ± 1.28	2.41 ± 1.27	0.917
Emergency room visits for asthma	6.87 ± 12.61	11.00 ± 14.59	0.090
Number of hospitalizations within the last 12 months	0.68 ± 1.71	1.03 ± 2.79	0.344
SCS use within the last 12 months	5.07 ± 9.76	9.47 ± 14.39	0.089
FEV_1_/FVC	0.11 ± 7.95	3.24 ± 9.72	0.073
FEV_1_ lt	0.06 ± 0.34	0.09 ± 0.48	0.725
FEV_1_ (% of predicted)	4.22 ± 13.19	3.87 ± 14.03	0.897
FVC lt	0.10 ± 0.37	0.17 ± 0.49	0.419
FVC (% of predicted) 5.13 ± 13.96 11.86 ± 24.65 0.174			

**Figure 1:**
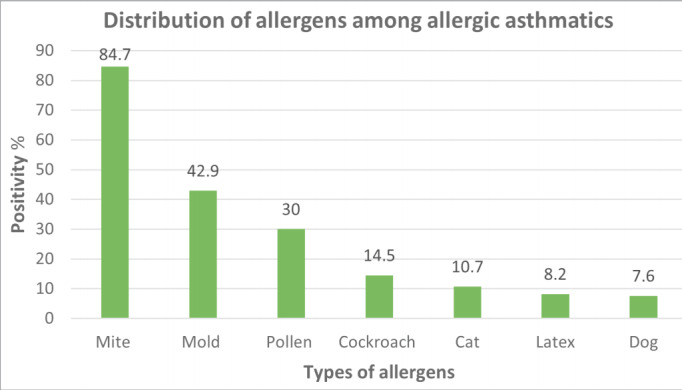
Ultrasound view of rectus femoris (RF) and vastus intermedius (VI) muscles in B mode.

Other than symptom control, omalizumab also has a positive effect on the quality of life and reduces exacerbation and hospital admission rates. Sözener et al. found a noticeable decrease in the number of exacerbations and hospitalizations in severe nonallergic asthmatic patients who received omalizumab (8^4^). Campo et al. also found a significant decrease in non-severe exacerbations, unplanned visits to primary care and specialists, and days of school/workplace absenteeism in severe non-atopic asthmatics one year after omalizumab ( 9 ). In a Spanish real-world, multicenter study, severe exacerbations significantly decreased in year one and year two after omalizumab treatment in both allergic and non-atopic severe asthmatics and results were not significantly different between the two groups ( 11 ). Our study is consistent with the reported findings where we observed significant decreases in the number of ER visits, hospitalizations, and steroid use for asthma exacerbations in both allergic and non-atopic severe asthmatics.

Another significant finding from our study was an increase in FEV_1_, FEV_1_%, FVC, and FVC% values with omalizumab treatment in the allergic group; with only FVC% increasing significantly in the nonatopic group (p< 0.05). Similarly, Sözener et al. and Campo et al. found a significant increase in FEV_1_% in the first year after omalizumab treatment in nonatopic severe asthmatics ( 8, 9 ). On the other hand, de Llano et al. found improved FEV_1_ at four months, one year, and two years in both allergic and non-atopic groups between initial and follow-up measurements, however, only allergic asthmatics had significant FEV_1_ increase ( 11 ).

Previous studies have shown that peripheral blood eosinophilia may be a key marker of the clinical response to omalizumab. In the INNOVATE study, omalizumab produced a greater reduction in exacerbation rate in patients with higher versus lower baseline eosinophil count ( 12 ), also a recent post-hoc analysis of two clinical studies has shown a greater reduction in exacerbation rate with omalizumab in patients with higher versus lower eosinophil count. Different from these studies, Humbert et al. found that- omalizumab response in patients with severe allergic asthma did not vary with blood eosinophil count; omalizumab appeared to be as effective in patients with “high” eosinophils (≥300 cells/µL-1) as in those with “low” eosinophils (<300 cells/µL-1) in the STELLAIR study ( 13 ). We did not compare groups of participants based on low and high eosinophil counts, as our study was not powered for this analysis. Decreased peripheral eosinophil counts were also noted in the literature with omalizumab treatment, as revealed in a pooled analysis of data from five randomized controlled trials, where  Massanari et al. discovered that post-treatment eosinophil counts were considerably lowered in the treatment group ( 14 ). Similarly, Türk et al. found decreased eosinophil counts with omalizumab treatment in allergic asthmatics but the difference was not statistically significant ( 15 ). In the current study, we found a significant decrease in eosinophil count after omalizumab treatment in our allergic asthmatics whereas non-atopic asthmatics had no significant decrease in eosinophil count. According to our findings, independent from eosinophils decrease both groups had similar decreases in symptomatic scores and exacerbation rates.

A glucocorticoid-sparing effect of omalizumab has been reported in several studies that included patients with severe allergic and non-allergic asthma ( 8, 16, 17, 18, 19 ). In our study, the mean daily SCS dose significantly decreased in allergic groups whereas non-atopics had a non-significant decrease. A possible explanation for this could be the lower number of patients on SCS.

Lommatzsch et al. proposed two possible explanations for the unpredicted effects of omalizumab in non allergic asthma. The first hypothesis suggested that these apparently non-atopic patients could be sensitized to unidentified allergens, resulting in a local airway-limited sensitization, while the second was based on the potential ability of omalizumab to restore the antiviral protective action of dendritic cells, which lost their ability to produce interferon upon FcεRI activation by allergen ( 20 ). Two randomized, placebo-controlled studies compared the change from baseline FcεRI expression on plasmacytoid dendritic cells (pDC2s), bronchial mucosal IgE+ cells, and blood basophils, respectively, in patients with non-atopic asthma after 14-16 weeks of treatment with omalizumab. Omalizumab significantly decreased the expression of FcεRI on plasmacytoid dendritic cells (pDC2s), bronchial mucosal IgE+ cells, and blood basophils in both studies but there was no correlation between these changes and clinical parameters. We agree with the study’s authors that larger trials are needed to fully understand the therapeutic effect of omalizumab in individuals with severe non-atopic asthma ( 7 , 21 ).

### Limitations

Our study has some significant limitations. First of all, it is a study retrospective, and all data were collected at baseline. We did not have the opportunity to obtain findings from repeated measures during the course of omalizumab treatment, therefore we could not examine patients’ clinical parameters and GINA scores year by year; only the first and last measurements could be evaluated. The second drawback was the small number of participants and the lack of a control group, which likely limited the analysis of demographic and clinical characteristics associated with therapy response.

Another drawback stems from the assumption that the absence of confirmed specific IgE antibodies and/ or positive SPT results means that an asthmatic can be categorized as non-atopic with certainty. Indeed, because the number of potential allergenic agents is far greater than the existing diagnostic tests can detect, false-negative results  are  likely to be obtained  ( 22 ). Furthermore, atopy is an age-related condition that diminishes over time ( 23 ). Since the non-atopic asthmatics in our study were significantly older, it is possible that they had previously been allergic. However, the SPT has the highest positive predictive value and is the most extensively used approach for allergy diagnosis.

## CONCLUSION

In conclusion, anti-IgE therapy can be a viable treatment option for non-atopic severe asthma. Our findings showed improvements in symptoms as well as objective indicators such as the number of exacerbations and hospitalizations, as well as lung function, which supports previous research. Further prospective placebo-controlled studies using adequate techniques are required to definitively establish the role of omalizumab in this setting.

## Ethical Committee Approval

The study was approved by Uludağ University Faculty of Medicine Clinical Research Ethics Committee (Decision no: 2020/10- 04, Date: 10.06.2020).

## Conflict of interest

The authors declare that they have no conflict of interest.

## AUTHORSHIP CONTRIBUTIONS

Concept/Design: DE, ME, FEG

Analysis/Interpretation: FEG, ME

Data acqusition: DE, FEG, GP

Writing: All of authors

Clinical Revision: DE, ME, GP

Final Approval: DE, ME, FEG
